# Analysis and Construction of the User Characteristic Model in the Adaptive Learning System for Personalized Learning

**DOI:** 10.1155/2022/5503153

**Published:** 2022-10-10

**Authors:** Xuekong Zhao, Shirong Long, Defa Hu

**Affiliations:** ^1^Education Technology and Information Management Center, Guangxi College of Education, Nanning 530023, China; ^2^Hunan University of Technology and Business, Changsha 410205, Hunan, China

## Abstract

Adaptive Learning System (ALS) is a supportive environment, which dynamically provides learners with services that can satisfy their demand for personalized learning in accordance with the differentiation of their individual traits. At present, study on ALS is still in the exploratory stage, and there are still many fields that deserve to be studied thoroughly. User characteristic model is the foundation and core of ALS and the key to the implementation of intelligent and personalized recommendation service. Based on this, this paper intends to analyze learners' characteristics in ALS through several dimensions, such as basic information, interest, preference, cognitive level and learning style, through which learners' user characteristic model is established. In the end, ALS, which supports the function of personalized recommendation, is implemented based on this model. It is suggested by the result of the simulation experiment that ALS, which is developed through this model, demonstrates a satisfying effect in recommendation, and it can dynamically recommend appropriate learning resources in accordance with learners' personalized demands through which learners' quality and efficiency of learning can be effectively enhanced to a certain extent.

## 1. Introduction

With the rapid development of Internet information and communication technology, E-learning, which is based on digitalized network, has gradually become an important learning style in the information era. Since E-learning demonstrates many advantages in timeliness, convenience, interaction, and resource-sharing, it makes learning activities more flexible, open, and liberal, and becomes quite popular among an increasing number of scholars. However, it is suggested by survey data that the current implementation of E-learning has not achieved a satisfying learning effect. This can be mainly attributed to the fact that many E-learning systems provide modularized or fixed functions and learning resources, and fail to provide accurate and superior learning resources in accordance with learners' demand for personalized learning. Consequently, there are such phenomena as poor quality and efficiency in learning, which leads to unsatisfying effect in the practical application. Hence, how to build an E-learning system to support personalized learning, and provide better and further services for users' learning has attracted extensive attention of many of researchers.

At present, many researchers have implemented a great deal of exploratory research work in certain fields, such as intelligent tutoring system, adaptive learning system, personalized virtual learning environment, and E-learning personalized recommendation technology. They have been making attempts to explore an optimal personalized solution to E-learning system through theoretical and practical research. Researchers, who hold the objective of making E-learning system more intelligent and personalized, and enabling it to dynamically recommend appropriate learning resources based on the accurate “understanding” of current learners' personalized demands, and further implementing the effect of systematic and personalized learning, have proposed their own viewpoints from different perspectives. Voiskovskii et al. [1] invented a method which can improve the algorithms in navigation systems, and they analyzed the configuration of the hardware and software in the prototype integrated navigation system. Ghadirli and Rastgarpour [2] introduced a simple E-learning system which determines learning style and characteristics of learners by a questionnaire and then makes learners' model. Kaziev and Glukhova [3] analyzed key methods, approaches, and technologies of adapting and intellectualizing web-learning, and they introduced a special model of adaptive learning decision-making. Alghofaily and Ding [4] took into consideration the dataset features in the QoS-based service recommendation process, and then used a meta-learning algorithm to incorporate dataset features in the recommendation process and study the use of different machine learning algorithms. Rafiq et al. [5] introduced the possibility of disaggregating query/question information in E-learning system online lectures or course recommendations, and then proposed a method for improving the classification of action verbs to a more accurate level in E-learning system. Sridharan et al. [6] invented an adaptive learning management system, which can create a customized course for every student based on their level of knowledge, preferred mode of learning, and continuously updating the course based on their learning speed. However, for accurate and personalized recommendation of learning resources, Tarus et al. [7] proposed that learner's context and sequential access patterns should be incorporated into the recommender system, and then introduced a hybrid recommendation approach for recommending learning resources. In addition, an idea of case-based reasoning has been adapted suitable for web page recommendation. Bhavithra and Saradha [8]suggested that apply weighted association rule mining to recommendation system for enhancing its accuracy. Collaborative filtering is an important approach to build a recommendation system. Ambulgekar et al. [9] introduced the recommendation technology of collaborative filtering and believed that this technology can be utilized in E-learning system to acquire learners' feedback information to learning resources, through which a personalized recommendation system can be implemented, etc.

Adaptive Learning System (ALS) is a personalized E-learning system solution. It can use data analysis technology to judge the characteristic attributes or behavioral tendencies of current learners in real time, and then use relevant teaching strategies, recommendation technologies, algorithms, and other technical means to dynamically provide learners with appropriate learning paths or learning resources to meet their personalized learning needs. For example, when learners prefer to browse video resources, the system will recommend more video resources for learners; when learners' cognitive level is high, the system will recommend some resources with higher difficulty level for learners. At present, a few achievements on the research of ALS are mostly concentrated on recommendation technology, recommendation algorithm, system modeling, adaptive engine, data mining, and cluster analysis. Specifically speaking, the commonly utilized recommendation technology mainly includes content-based recommendation, collaborative filtering recommendation, association rule recommendation, and integrated recommendation [10]. In terms of ALS modeling, there are also some typical representative achievements: Babori et al. suggested that the frameworks model of ALS should be composed of three components: learner model, domain model, and adaptation model, and then invented a framework based on self-regulated learning (SRL) [11]. Vesin et al. [12] considered that an intelligent tutoring system should provide smart and interactive content, personalization options, adaptive features, and then they presented the interactive learning analytics component developed in ProTu, so as to deeply discuss the key technologies of ALS recommendation.

By comparing and analyzing the above theoretical-practical research achievements, it is clear that the achievements can provide reference for our subsequent research in relevant fields. At the same time, it is also found that although many researchers have proposed various ideas for building the ALS system framework, their research results or conclusions are still being explored and improved. For example, some researchers focus on recommendation algorithms, adaptive engines, or data mining, but their thinking on the user model of ALS system is not comprehensive and in-depth enough, which leads to the unsatisfactory recommendation effect of the ALS system [13]. Based on this, we believe that it is necessary to conduct a comprehensive and in-depth study on the user feature model of the ALS system. In this study, we will focus on more comprehensive analysis and construction of user feature model from multiple dimensions such as user basic information, interest preference, cognitive level, and learning style, so as to explore a new approach to personalized recommendation in the ALS system. In order to carry out in-depth research, we will model and analyze the attribute structure, behavior characteristics, and construction process of the user model of the ALS system based on other research results, and we will propose a more comprehensive user feature model structure. On this basis, we focus on exploring the representation mode, matching relationship, and computational reasoning process of user feature model from multiple dimensions, and propose the multiple-tuple representation method and calculation formula of user basic model, interest preference model, cognitive level model, and learning style model. Finally, a simulation experiment scheme is proposed to verify the personalized recommendation effect of the research results. It is suggested by the result of the simulation experiment that the ALS system, which is developed through this model, demonstrates a satisfying effect in recommendation. Furthermore, it can dynamically recommend appropriate learning resources in accordance with learners' personalized demands, which can effectively enhance learners' quality and efficiency of learning to a certain extent.

## 2. Analysis of the User Characteristic Model for Personalized ALS

In accordance with the characteristics and demands of personalized learning, ALS users generally include learners and resource managers. Specifically speaking, learners are the acquirer of learning resources in ALS and the main object of the implementation of personalized recommendation service; managers are mainly in charge of the management and update of ALS learning resources, and this position is generally taken by system managers or class teachers. During the process of E-learning, there are significant differences among individual learners in knowledge base, disciplinary background, age, gender, learning style, and personal abilities, which will further lead to constant changes and development of the learning process and the grasp of knowledge. In consideration of these, whether the system can accurately acquire and analyze current learners' status and information is the premise of the recommendation of personalized learning materials to current learners and the implementation of intelligence and adaptability. Based on this, a user feature model for ALS learners is mainly established and analyzed in this paper.

User feature model is a specific representation of system users in the computer field. In the ALS system, the user feature model plays a fundamental role. It is not only the core component of the system to achieve intelligence and adaptability but also an important basis for the system to accurately recommend personalized resources. When the ALS system is running, it will generally analyze and judge the characteristic attributes of the current user, and then use them as the basis for user learning needs to dynamically recommend personalized learning paths or learning resources. Generally, user feature model has many characteristics of learning behavior attributes, so many researchers have explored it based on the perspective of learner model. For example, there are many viewpoints on the description of learner model: Sweta and Lal [14] believed that learners' learning style and behaviour to provide suitable learning paths are important to the accuracy of ALS recommendation, so they suggested that learning style and learning behavior should be able to be dynamically detected in ALS, and then constructed a personalized adaptive learner model (PALM). By experimental research, Fatahi [15] proposed that personality and emotion are important parts of learner feature model, and play important roles in the adaptive learning system. Lwande et al. [16] believe that learning style is very important for the definition of learner model. Through learning behavior log analysis, learners' style preference can be predicted, which can be used as the basis for learning system recommendation. By the analysis of the abovementioned viewpoints, it is believed that the learner model is a mathematical model, which is established to present learners' individual traits, such as personal information, knowledge base, and cognitive structure, with the objective of reflecting learners' learning through the E-learning system. In the practical application, since ALS attaches much importance to the development of current learners' cognitive dimension, learner model's accurate acquisition of learners' understanding of knowledge and content is of great importance. Learners' cognitive state is generally their grasp of learning resources and an important constituent part of the learner model.

**Figure 1:**
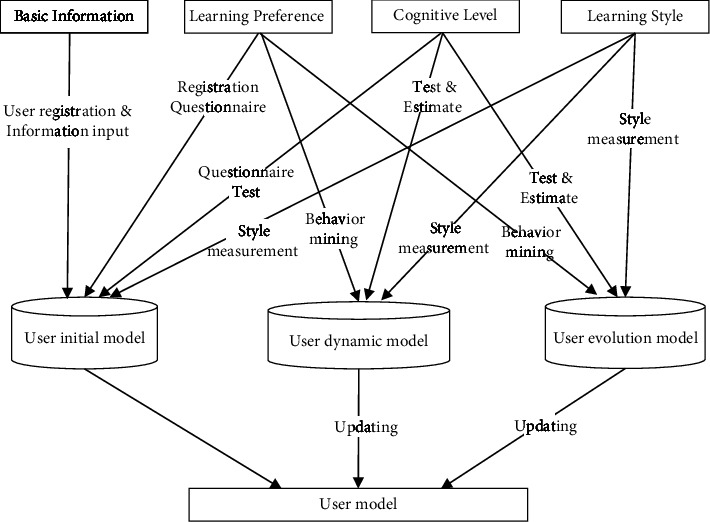
Construction of learners' user model.

Based on the consideration of the general attribute characteristics, behavior operation and learning needs of learner users in the E-learning environment, and on the basis of reference to other research results, this study analyzes the IMS LIP modeling standard of learner information package in the international integrated management system, and then proposes the basic structure of user feature model of adaptive learning system from a more comprehensive perspective. The user feature model mainly includes four dimensions: basic information, learning preference, cognitive level, and learning style, which can basically meet the general needs of personalized recommendation in the ALS system. Of course, in the future research work, we will continue to enrich and improve the model in this field to provide more scientific and comprehensive research results. In the user feature model, basic information features mainly represent some static information of students, such as student number, name, gender, age, major, learning experience, etc. Interest and preference is mainly utilized to describe learners' preference of the type of learning resources. As the key to the learner model, learning style and cognitive level are utilized to store learners' dynamic information and jointly represent learners' state. After sorting out the personalized recommendation function of the ALS system and its implementation technical scheme, we will explore to build a learner model framework with dynamic updating effect. Please see the modeling process from Figure 1.

It can be seen from Figure 1 that the construction of a learner model is a dynamic updating process, which mainly includes three links: user initial model, user dynamic model, and user evolution model. Among them, the user initial model is mainly used to represent the basic-static information of learners, such as student number, name, major, etc. User dynamic model is mainly used to represent the dynamic change characteristics of learners in the learning process, such as learning preference, cognitive level, learning style, etc. User evolution model is mainly used to represent the change of learners' learning behavior, and its analysis results can be used as an important data source for updating user dynamic model. In the ALS system, users' basic information, interest preferences, cognitive level, and learning style characteristics can be stored in the form of two-dimensional relationship tables or log files. For example, the user's basic information can be stored in the user table, and the user's learning behavior record can be stored in the log file. When the ALS system is running, the user's ID index can be used to query the current learner's basic personal information, and the data mining technology can also be used to analyze and obtain the latest learning state characteristics of the current learner from the user's behavior log file, such as learning preference, cognitive level, etc. According to the dynamic construction process of the user model, after the ALS system obtains various user feature information, the system will usually use the program algorithm to dynamically update the user model in real time, and use this as the basis for subsequent judgment and analysis of user feature status, and then recommend personalized learning materials for them.

**Figure 2:**
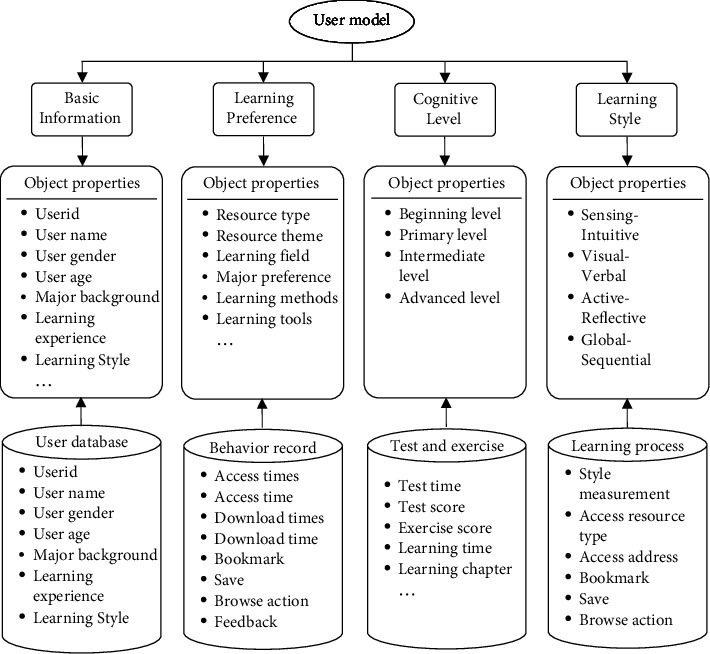
User behavior characteristic-based user model.

In the E-learning environment, each user often has different behavior activities in the learning process. These behavior activities can reflect varied implicit information such as the learning style characteristics, cognitive level, interest preference, etc. For instance, if a user browses a certain knowledge point for a long time during the learning process, it indicates that this user has a high degree of interest or demand for such knowledge point content. If a user frequently accesses, downloads, or saves a certain type of resource during the learning process, it indicates that the user prefers this type of learning resources. Thus, it can be seen that it is very important to start with the behavior activities of users in the E-learning environment, explore the individual behavior characteristics of users hidden behind these behavior activities, and further explore their value and build user models. On this basis, an attempt is made to further describe user behavioral model from four dimensions, namely, basic information, learning preference, cognitive level, and learning style. Please see the constructed user model from Figure 2 [17, 18].

The user behavior characteristic model (Figure 2) shows that the modeling of users' basic information dimension is acquired directly through the user database. User's learning preference model includes resource type, resource theme, learning field, major preference, learning methods, learning tools, etc. It excavates data through behavior record during user's E-learning. User's cognitive level model, which includes beginning level, primary level, intermediate level, and advanced level, is acquired from testing and exercise database. Learning style model, which includes four properties as sensing-intuitive, visual-verbal, active-reflective, and global-sequential, can be comprehensively assessed and acquired through the result of user's learning style measurement, the type of access resources, and the excavation of browsing behavior. Specifically speaking, user behavior characteristic includes access times, access time, download times, download time, add bookmark, save, browse action, and feedback. User's browsing behavior mainly includes the times of clicking the mouse, scrolling the scroll bar, and pressing the up and down button. Under normal circumstances, more operation behaviors during webpage-browsing can indirectly reflect that the user is more interested in this web page, for example, if a user shows behaviors like frequently accessing a website, saving a website, adding a bookmark, and downloading resources, this user's interest in this web page can be confirmed [19, 20].

After the above analysis of the dimensions of constructing a user characteristic model, relevant matching rules, expressions, and algorithms can be utilized to analyze user's demand for learning resources, and confirm user's characteristic model in ALS. The specific methods of construction will be described in the next article.

## 3. Methods of Constructing User Characteristic Model for Personalized ALS

### 3.1. Basic User Model

Substantively speaking, personalized recommendation forwards and presents appropriate resources in accordance with the current users' personalized demands. Learner's user model is the foundation of ALS modeling. When constructing the user basic model, the user's characteristic attribute may include static information such as name and gender, and also dynamic information such as interest preference or learning experience. The user model can be constructed by the following method [21, 22]:

(1) $$\begin{array}{c} UserModel=\left(BasicInformation,LearningPreference,CognitiveLevel,LearningStyle\right)\text{.} \end{array}$$

In expression (1), “BasicInformation” represents user's basic-static information, which includes student no., name, gender, age, professional background, learning experience, user name, password for login, etc. “LearningPreference” represents user's learning preference, which is represented through user's interest in learning resources. “CognitiveLevel” represents user's current cognitive level or learning ability. “LearningStyle” represents user's learning style. Learning Preference, Cognitive Level, and Learning Style, which store user's dynamic information, are the key to ALS modeling and the implementation of personalized recommendation. Furthermore, real-time dynamic update is realized in the value with the changes of the status of E-learning. Next, an emphasis will be placed on the elaboration of the specific modeling method for Learning Preference, Cognitive Level, and Learning Style.

### 3.2. User Interest and Preference Model

User interest model is an integrated description of user's information and demand during the learning after logging into the system, which reflects the current user's preference or interest tendency for a certain learning content. For example, if the user's browsing time or download times for a learning resource increases, it indicates that the user prefers the learning resource. User interest information can generally be obtained by user active description and user behavior record mining. In order to ensure that the ALS system can more accurately support personalized learning, based on the thinking of the dynamic updating process of the user feature model, we believe that it is an important way to build the current user's interest preference model based on user behavior records. Here, we will conduct a comprehensive modeling and analysis of all learning behaviors related to learners' interests and preferences in the ALS system, such as the access times, access time, download times, bookmark actions, mouse events, etc., to ensure the integrity and scientific nature of the data recording user behavior characteristics. After the ALS system records the user behavior preference information in the database table or log file, the system will use the user learning preference model as the basis to obtain more accurate interest values by mining the user's implicit learning behavior, and then recommend appropriate personalized learning resources. In this section, we will focus on how to calculate the user's interest in the current learning resources based on the user's learning behavior characteristics, and build its interest preference model.

In accordance with the above analysis, the calculation of user's interest mainly takes the attention to the web page or learning resources as the main basis, which can be calculated and reflected through user's behavior characteristic. The more attention that a user pays to the learning resources, the more interest he or she holds in the learning resources. Meanwhile, it is also a reflection of the user's increasing demand for the learning resources. With the purpose of calculating user's interest, the mode of tuple is adopted to construct a calculation model of user's attention. The user interest model and the calculation method of its interest degree are given as follows [23, 24]:

(2) $$\begin{array}{c} InterestDegree\left(n\right)=\left\{\langle R_{1},A_{1}>,<R_{2},A_{2}>,\cdots ,<R_{i},A_{i}>,\cdots ,<R_{p},A_{p}\rangle \right\}. \end{array}$$

In expression (2), *R*_*i*_ represents the collection of webpages or resources for learning, *A*_*i*_ represents user's attention to *R*_*i*_, and *p* is the quantity of the collection of webpages or resources for learning, *i*=1,2,3, ⋯*p*. User's attention to the resources can be acquired through the accurate feedback information and access pattern. Please see the method of presenting the degree of attention in the following expression:

(3) $$\begin{array}{c} A_{i}=A\left(U,R_{i},T_{i}\right)\text{.} \end{array}$$

In expression (3), 0 ≤ *A*_*i*_ ≤ 1, *U* represents learner user, *R*_*i*_ represents the learning resources being accessed, *T*_*i*_ represents the type of user's behavior characteristic, *i* represents the number of learning resources, and *i*=1,2,3, ⋯*p*, A_i_ represents user (*U*)'s attention to the learning resources *R*_*i*_. In accordance with the above analysis of user's behavior characteristic, the value range of *T*_*i*_ in expression (3) is *T*_*i*_∈{Access times, Access time, Download times, Bookmark, Save, Browse action, Feedback,…}. Access times represent the times of accessing webpages or learning resources; Access time represents the time of accessing webpages or learning resources; Download times represents the times of downloading learning resources; Bookmark represents the behavior and action of adding learning webpages to the bookmark; Save represents the behavior and action of saving learning webpages; Browse action represents the behavior and action of browsing learning webpages, such as scrolling the mouse, clicking the mouse, moving forward and backward, etc.; Feedback represents user's feedback to the given resources.

In accordance with the above user's behavior characteristic model, data matching and switching is needed in the model of attention degree, namely, expression (3), so that user's interest in learning resources can be calculated. With regard to the model of attention degree, the rules of data matching and switching are set up as follows: if a user downloads resources, adds webpages to the bookmark, and saves webpages, it suggests that this user holds a higher degree of expectation and interest in the learning resources presented through the webpages, and under this circumstance, the user's degree of interest is set as 1; if none of these behaviors or actions happen, the user's interest in this kind of learning resources can be calculated through other behaviors. Please see the method of calculation from the following expression:

(4) $$\begin{array}{c} ID=\left\{\begin{array}{cc} 1\text{,} & T_{i}\in \left\{Downloa\;dB\;ookmarkSave\right\}\text{,}\\ f_{1}\left(ats,dts\right)\text{,} & T_{i}\in \left\{Access\;timesDownloa\;dt\;imes\right\}\text{,}\\ f_{2}\left(at,sc,cc\right)\text{,} & T_{i}\in \left\{Access\;timeBrowseaction\right\}\text{.} \end{array}\right. \end{array}$$

In expression (4), ID represents the degree of interest; *T*_*i*_ represents the type of user's behavior characteristic, {Downloa d, Bookmark, Save} represents the collection of a group of behaviors that the user might show during webpage-browsing, including downloading resources, adding webpages to a bookmark and saving learning webpages; *ats* represents the times of accessing webpages or learning resources; *dt* *s* represents the times of downloading resources; *at* represents the time of accessing webpages, namely, the learning time; *sc* represents the times of scrolling the scroll bar when the user is browsing webpages; *cc* represents the times of mouse-clicking when the user is browsing webpages; *f*_1_(*ats*, *dts*) represents the mapping function from *ats* and *dts* to *RD*; *f*_2_(*at*, *sc*, *cc*) represents the mapping function from *at*, *sc*, and *cc* to *ID* [23, 24].

As for the mapping function *f*_1_(*ats*, *dts*) in expression (4), since user's times of accessing resources *ats* and times of downloading resources *dts* cannot objectively demonstrate user's interest in the resources, the average times of accessing and downloading resources need to be quoted to evaluate the current user's interest degree. Therefore, the function *f*_1_(*ats*, *dts*) can be replaced with expression (5). Please see the specific expression as follows:

(5) $$\begin{array}{c} f_{1}\left(ats,dts\right)=\left\{\begin{array}{cc} 1\text{,} & \left(ats>\bar{ats_{0}}\;,dts>\bar{dts_{0}}\right)\text{,}\\ \frac{\alpha ats}{\bar{ats_{0}}}+\frac{\beta \;dts}{\bar{dts_{0}}}\text{,} & \left(ats<\bar{ats_{0}}\;,dts<\bar{dts_{0}}\right)\text{.} \end{array}\right. \end{array}$$

In expression (5), *ats* represents the times of accessing webpages or learning resources; *dts* represents the times of downloading resources; $\bar{ats_{0}}$ represents the average times of accessing webpages or learning resources; $\bar{dts_{0}}$ represents the average times of downloading resources; *α* and *β* represents weighing parameters. The modes of calculating $\bar{ats_{0}}$ and $\bar{dts_{0}}$ are listed, respectively, as follows:

(6) $$\begin{array}{c} \bar{ats_{0}}=\frac{ats\_num}{usernum}\bar{dts_{0}}=\frac{dts\_num}{usernum}\text{.} \end{array}$$

In expression (6), *ats*_num represents the total times of accessing learning webpages or resources; user_num represents the total number of users in the system; *dts*_num represents the total times of accessing learning webpages or resources. When $\alpha ats/ats_{0}+\beta dts/\bar{dts_{0}}$≥1, the value of *f*_1_(*ats*, *dts*) is set as 1; when 0<*αats*/*ats*_0_+*βdt* *s*/*dts*_0_<1, the value of *f*_1_(*ats*, *dts*) is $\alpha ats/ats_{0}+\beta dts/\bar{dts_{0}}$.

As for the mapping function *f*_2_(*at*, *sc*, *cc*) in expression (4), the time of accessing webpages *at* is under the influence of various factors, such as reading speed, length of content and form of expression on the webpages. More content generally means longer accessing time; more video resources also mean longer accessing time. In addition, since the mode of reading pictures and video resources manifests substantial difference from the mode of reading texts, it is quite difficult to simply quantify the time of accessing webpages *at*. In consideration of this, the text resource is emphatically taken as an example in this paper, and the access time ratio is adopted to eliminate the influence brought about by the length of content of user's own factors. Please see the method of calculation from the following expression:

(7) $$\begin{array}{c} atr=\frac{at}{\bar{at_{0}}}=\frac{at\times trs}{cl}\text{.} \end{array}$$

In expression (7), *atr* represents access time ratio; *at* represents user's access time; $\bar{at_{0}}$ represents the average access time, and the mode of calculation is $\bar{at_{0}}=cl/trs$. *cl* represents content length; *trs* represents user's text reading speed, and it can adopt the average standard value of reading speed.

In accordance with user's access behavior and habit, the maximum and minimum threshold value of the average access time is set as *atr*_min_ and *tr*_max_, respectively. If the abovementioned function *f*_2_(*at*, *sc*, *cc*) is replaced by *f*_2_(*atr*, *sc*, *cc*), a piecewise function can be achieved. Please see the method of expression as follows:

(8) $$\begin{array}{c} f_{2}\left(atr,sc,cc\right)=\left\{\begin{array}{cc} 0\text{,} & \left(atr<atr_{\min}\right)\\ atr\text{,} & \left(atr_{\min}<atr<atr_{\max}\right)\text{,}\\ f_{3}\left(sc,cc\right)\text{,} & \left(atr>atr_{\max}\right)\text{.} \end{array}\right. \end{array}$$

Expression (8) suggests that when *atr* < *atr*_min_, it signifies that the user spends very little time accessing the webpages, and the interest degree can be recorded as 0; when *atr*_min_ < *atr* < *atr*_max_, it signifies that the user spends a normal length of time accessing the webpages, and the interest degree can be acquired through normal calculation, namely, *f*_2_(*atr*, *sc*, *cc*)=*atr*; when *atr* > *atr*_max_, it signifies that the user spends a lot of time accessing the webpages, and the state of access might be abnormal, for example, the user forgets to close the web page after clicking open. Under this circumstance, another behavioral function *f*_3_(*sc*, *cc*) can be adopted to acquire the interest degree. Function *f*_3_(*sc*, *cc*) mainly involves such behavioral characteristics as scrolling the scroll bar and clicking the mouse. Under general circumstances, the times of scrolling the scroll bar and clicking the mouse should be directly proportional to the length of the content. In other words, if there is much content on the web page, the times of scrolling the scroll bar and clicking the mouse will be more. With the purpose of calculating the user's interest degree more accurately and objectively, the function *f*_3_(*sc*, *cc*) is expressed through piecewise function. Please see the mode of expression from the following expression:

(9) $$\begin{array}{c} f_{3}\left(sc,cc\right)=\left\{\begin{array}{cc} 0\text{,} & \left(f_{3}{\left(sc,cc\right)}^{\prime }<0\right)\text{,}\\ f_{3}{\left(sc,cc\right)}^{\prime }\text{,} & \left(0\leq f_{3}{\left(sc,cc\right)}^{\prime }\leq 1\right)\text{,}\\ 1\text{,} & \left(f_{3}{\left(sc,cc\right)}^{\prime }\geq 1\right)\text{.} \end{array}\right. \end{array}$$

In expression (9), *f*_3_(*sc*, *cc*)′ is the binary regression of user's behavior characteristic of scrolling the scroll bar and clicking the mouse during webpage-browsing, and its method of calculation is *f*_3_(*sc*, *cc*)′=*α* × *sc*/*cl*+*β* × *cc*/*cl*+*γ*/*cl*. *sc* represents the times of scrolling the scroll bar; *cc* represents the times of clicking the mouse; *cl* represents the length of content; *α*, *β*, and *γ* represent weighting parameters that are about to be estimated.

### 3.3. User Cognitive Level Model

User cognition level indicates the ability of individual users to understand and process information. In the ALS system, the user's cognitive level reflects the user's mastery of the learning content, which provides a decision-making basis for the ALS system to recommend learning materials of various difficulty levels. In order to make the ALS system determine the current cognitive level of users more accurately, it is usually possible to conduct a comprehensive evaluation from multiple dimensions such as learners' user test scores, test duration, learning duration, and learning behavior. For example, if the user's current unit test score is very good, it indicates that the user's cognitive level of the unit knowledge is high. If the user's current unit test lasts for a long time, it indicates that the user's cognitive level of the unit knowledge is not high enough. When the ALS system is running, it will dynamically obtain the user's current cognitive level information, update the user's cognitive level model in a timely manner, and then match the learning resources in the system on this basis to recommend learning materials suitable for users. An attempt is made to adopt the method of four-level classification to determine the user's cognitive level based on the test performance. The four levels are the beginning level, primary level, intermediate level, and advanced level, respectively. And, the sectional range is set as {[0–59], [60–69], [70–89], [90–100]} accordingly, and the corresponding cognitive level is recorded as {1, 2, 3, 4}. Thus, user cognitive level model can be defined as follows [21, 25]:

(10) $$\begin{array}{c} CognitiveLevel\left(U\right)=\left(U,CL_{i}\right)\text{.} \end{array}$$

In expression (10), *U*represents learner user; *CL*_*i*_ represents user's cognitive level, and it is under the range of *CL*_*i*_∈{1, 2, 3, 4}.

Likewise, the grade of difficulty also needs to be set up to implement the function of recommending learning materials of different grades of difficulty in accordance with user's cognitive level. In consideration of this, we hereby divide the grade of difficulty into four grades, namely, easy, relatively easy, average, and difficult. The grade of difficulty is recorded as {1, 2, 3, 4} accordingly. Thus, the level model of learning materials can be defined as follows:

(11) $$\begin{array}{c} CognitiveLevel\left(R\right)=\left(R,DL_{i}\right)\text{.} \end{array}$$

In expression (11), *R* represents learning material; *DL*_*i*_ represents the level of learning material, and it is under the range of *DL*_*i*_∈{1, 2, 3, 4}. Under general circumstances, the value of *DL*_*i*_ is set up by the course teacher or system manager during the establishment of curriculum resources on the back-stage management web page.

After the above cognitive level and the grade of difficulty of the learning materials are confirmed, the screening of resources can be implemented through matching rules. Please see the specific method as follows:

(12) $$\begin{array}{c} CognitiveLevel\left(CL_{U},CL_{R}\right)=\left\{\begin{array}{c} CL_{R}X>0\\ CL_{U}X\leq 0 \end{array}\right.\left(X=CL_{U}-CL_{R}\right)\text{.} \end{array}$$

In expression (12), *CL*_*U*_ represents user's cognitive level, namely, the value of CognitiveLevel(*U*)=(*U*, *CL*_*i*_). *CL*_*R*_ represents the grade of difficulty, namely, the value of CognitiveLevel(*R*)=(*R*, *DL*_*i*_). CognitiveLevel(*CL*_*U*_, *CL*_*R*_) represents the matching degree of user (*U*)'s cognitive level and the grade of difficulty of learning materials *R*. If the learner's current cognitive level is higher than the difficulty level of the learning materials presented by the system, the personalized learning materials will be randomly recommended based on the system. On the contrary, if the learner's current cognitive level is lower than the difficulty level of the learning materials presented by the system, the personalized learning content that meets the cognitive ability of the users will be recommended based on the learner's current cognitive level.

To a certain extent, the length of online testing also reflects user's cognitive level. Hence, the length of online testing also needs to be taken into consideration during the assessment of the cognitive level. Longer length of online testing generally signifies lower cognitive level. On the contrary, if the length of online testing is shorter, it signifies higher cognitive level. Under this circumstance, the function *F*(*T*) can be adopted to manifest the relationship between user's cognitive level and the length of online testing [25, 26].

(13) $$\begin{array}{c} F\left(T\right)=\left\{\begin{array}{cc} 1\text{,} & T\leq T_{r}\text{,}\\ 1-\frac{T}{\left(T_{r}+T_{m}\right)}\text{,} & T_{r}<T\leq T_{m}\text{,}\\ 0\text{,} & T\geq T_{m}\text{.} \end{array}\right. \end{array}$$

In expression (13), *T*_*r*_ represents the normal length of testing; *T*_*m*_ represents the maximum length of testing. Expression (13) shows that when the length of testing is shorter than the normal length, it can be deemed that the user's cognitive level is higher, and the value is set as 1; on the contrary, when the length of testing is longer than the maximum length, it can be deemed that the user's cognitive level is relatively lower, and the value is set as 0. Likewise, the relationship between user's cognitive level and the length of online testing can be achieved. Please see the method of calculation as follows:

(14) $$\begin{array}{c} H\left(T\right)=\left\{\begin{array}{cc} 1\text{,} & T\leq P_{r}\text{,}\\ 1-\frac{T}{\left(P_{r}+P_{m}\right)}\text{,} & P_{r}<T\leq P_{m}\text{,}\\ 0\text{,} & T\geq P_{m}\text{,} \end{array}\right. \end{array}$$

In expression (14), *P*_*r*_ represents the normal length of learning a certain chapter or knowledge point; *P*_*m*_ represents the maximum length of learning.

In conclusion, the matching degree between the user and learning materials can be calculated through the user's cognitive level, the grade of difficulty of learning materials, and the length of online testing and learning. Please see the expression as follows:

(15) $$\begin{array}{c} CognitiveLevel\left(U,R\right)=\alpha CognitiveLevel\left(CL_{U},CL_{R}\right)+\beta F\left(T\right)+\gamma H\left(T\right)\text{.} \end{array}$$

In expression (15), CognitiveLevel(*U*, *R*) represents the matching degree between the user and learning materials, and greater value represents higher matching degree here. *α*, *β*, *γ* are weighing parameters that can be set up by course teacher (or system manager) through the system management web page.

### 3.4. User Learning Style Model

Learning style is one of the research fields of pedagogy and psychology. In recent years, it has also attracted the attention of computer science. Especially, in the aspect of adaptive personalized recommendation system, many researchers try to explore it deeply. Learning style generally refers to an inherent or biased characteristic pattern of individual learners' perception, acquisition, and processing of information in the learning process. It is an important embodiment of individual differences of learners. For example, some learners prefer to browse video learning resources, while others prefer to browse text learning resources, which reflects the different learning styles of learners. As for the types of learning styles, many researchers agree with the classification method of learning styles proposed by psychologist Felder et al., which divides learning styles into four categories based on the four dimensions of learners' perception, input, processing, and understanding of information: sensing-intuitive, visual-verbal, active-reflective, and global-sequentia (see Table 1). In the ALS system, the user learning style model is mainly used to represent the characteristics of the user learning style, which has an important reference basis for the system to recommend personalized learning paths or learning resources. In order to accurately represent the learning style model of learners in the ALS system, this study constructs learner personality attributes based on four dimensions of Felder's learning style model: perception, input, processing, and understanding, and each dimension is corresponding with two opposite style types, respectively. When the ALS system is running, it can extract the current user's learning style type from the user's learning style model, and match the user's learning style with the learning resources in the system for associated attributes, and then provide personalized learning services for the current user by recommending resources that match the user's learning style. For example, users of visual learning style will be mainly recommended for video or animation resources, while users of verbal learning style will be mainly recommended for text or audio resources. Next, we will focus on the representation method and construction process of user learning style model in the ALS system [19, 27, 28].

Based on Felder's four-dimensional learning style model, an attempt is made to adopt the form of tetrad to construct user learning style model. Please see the method of expression as follows [20]:

(16) $$\begin{array}{c} LearningStyle\left(U\right)=<D_{1},ls_{1}>,<D_{2},ls_{2}>,<D_{3},ls_{3}>,<D_{4},ls_{4}>\text{.} \end{array}$$

In expression (16), <*D*_*i*_, *ls*_*i*_ > (1 ≤ *i* ≤ 4) represents learner's value in a certain dimension under Felder's learning style, *D*_*i*_ represents the type of style value (*D*_*i*_∈{“Sensing-Intuitive,” “Visual-Verbal,” “Active-Reflective,” and “Global-Sequential”}), *ls*_*i*_ is fuzzy value (*ls*_*i*_∈[0, 1]), which represents the value of the dimension learning style *D*_*i*_.

With the purpose of matching the rules of the user's learning style and learning resource model, the correlation between the type of learning resources and learning style also needs to be taken into consideration in the establishment of the system model. In consideration of this, the following learning resource model is established:

(17) $$\begin{array}{c} ResourceMode\;l=\left(ResourceInformation,ResourceStyle\right)\text{.} \end{array}$$

**Figure 3:**
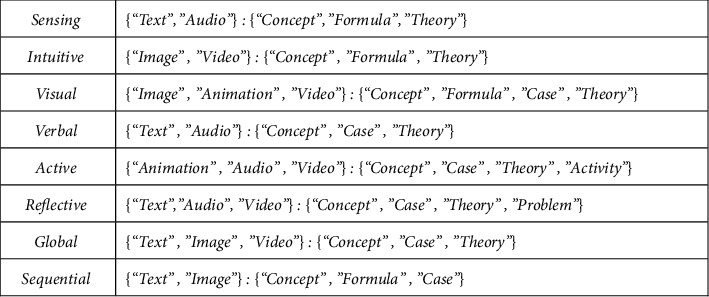
Matching relation between learning style and resource type.

In expression (17), ResourceInformation represents basic information of resources, such as name, size, uploader, time and path of uploading, etc.; ResourceStyle represents the type of decision style. ResourceStyle can be represented as ResourceStyle(*i*)={〈*rs*_*i*_, *rt*_*i*_〉} in the form of two-tuples. Specifically speaking, *rs*_*i*_ represents the media type, and *rs*_*i*_ ∈ {^″^*Text*^″^, ^″^Image^″^, ^″^Animation^″^, ^″^Au di o^″^, ^″^Vi de o^″^}; *rt*_*i*_ represents the strategy type, and *rt*_*i*_ ∈ {^″^Concept^^″^^, ^″^Formula^″^, ^″^Case^″^, ^″^Theory^″^, ^″^Problem^″^, ^″^Activity^″^}. For instance, ResourceStyle(*p*)={<^″^Image^″^, ^″^Case^″^>} represents that the decision style type of resource *p* is picture and case-oriented. After this, a resource model is established, Felder-Silverman style dimension can be taken as a reference to build a matching relation between learning style and resource model. Please see the rule of relation in Figure 3 [20, 29].

Figure 3 suggests the relation between learning resource type and learning style. It cannot accurately calculate the matching relation between learning style and resources through the method of quantitative processing. Hence, the matching relation between learning resource model and learning style needs to be further converted into an expression that is of mathematical significance. An attempt is made to construct the learning resource style model through the form of tetrad. Please see the specific method of expression as follows [20, 25].

(18) $$\begin{array}{c} ResourceStyle\left(R\right)=<D_{1},rs_{1}>,<D_{2},rs_{2}>,<D_{3},rs_{3}>,<D_{4},rs_{4}>\text{.} \end{array}$$

In expression (18), <*D*_*i*_, *rs*_*i*_ > (1 ≤ *i* ≤ 4) represents the value of knowledge object in a certain dimension under Felder's learning style, *D*_*i*_ represents the style type (*D*_*i*_∈{“Sensing-Intuitive,” “Visual-Verbal,” “Active-Reflective,” “Global-Sequential”}), the value of *rs*_*i*_ is −1 or 1. The value of *rs*_*i*_ is generally set up by course teacher or system manager in light of the matching rules of Figure 3 during the establishment of curriculum resources.

It can be inferred from expression (16) and expression (18) that the style matching degree of the learning and learning materials is decided by the sum of the numerical value on the diagonal line of the matrix multiplication of Learning Style and Resource Style.

The process of inference: tetrad expression (16) and expression (18) that are defined above are converted into 4 × 1 column matrix and 1 × 4 row matrix, respectively, and

(19) $$\begin{array}{c} LearningStyle\left(U,R\right)=LearningStyle\left(U\right)\times ResourceStyle\left(R\right)=\left(\begin{array}{c} ls_{1}\\ ls_{2}\\ ls_{3}\\ ls_{4} \end{array}\right)\times \left(\begin{array}{cccc} rs_{1} & rs_{2} & rs_{3} & rs_{4} \end{array}\right)=\left(\begin{array}{cccc} lrs\left[1,1\right] & \;lrs\left[1,2\right] & lrs\left[1,3\right] & lrs\left[1,4\right]\\ lrs\left[2,1\right] & lrs\left[2,2\right] & lrs\left[2,3\right] & \;lrs\left[2,4\right]\\ lrs\left[3,1\right] & \;lrs\left[3,2\right] & \;lrs\left[3,3\right] & lrs\left[3,4\right]\\ \;lrs\left[4,1\right] & lrs\left[4,2\right] & \;lrs\left[4,3\right] & lrs\left[4,4\right] \end{array}\right)\text{.} \end{array}$$

In expression 19, *LearningStyle*(*U*, *R*) is the product of two matrixes and a fourth-order matrix. A greater sum of the numerical value of the diagonal line signifies the higher matching degree between the learning and learning resources. This value can be achieved from

(20) $$\begin{array}{c} LearningStyle\left(U,R\right)=\left(ls_{1}-C\right)\times rs_{1}+\left(ls_{2}-C\right)\times rs_{2}+\left(ls_{3}-C\right)\times rs_{3}+\left(ls_{4}-C\right)\times rs_{4}. \end{array}$$

In expression (20), *C* is a constant, and it is the median of the data range of *ls*_*i*_(*ls*_*i*_∈[0, 1]).

## 4. Simulation Experiment and Discussion

### 4.1. Simulation Experiment Design

**Figure 4:**
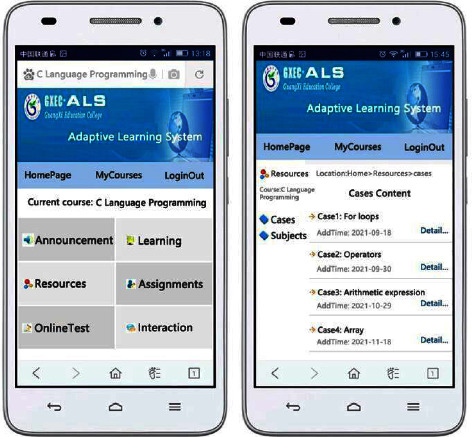
Effect of ALS course learning page via a mobile phone.

With the purpose of examining the validity of the research achievement, a user characteristic model is constructed based on UML modeling standard and XML + SQL Server database technology after the analysis and organization of the user characteristic model for personalized ALS. And then, NET Framework environmental development is utilized on this basis to implement the prototype of ALS. The development language mainly utilizes C#, C, VBScript, JavaScript, SQL query language, etc. In terms of the function of system implementation, the current ALS can provide different functions to students and teachers. It can not only satisfy the demands of general classroom teaching but also support students' online personalized and independent learning. When a student user logs in the system, he or she can select courses online, study courses, browse resources, submit assignment, take online tests, etc. When a teacher user logs in the system, he or she can examine and verify students who are selecting courses, manage curriculum resources, correct assignments, etc.  Figure 4 suggests the effect of course learning page when a student user utilizes the mobile phone to log in the system. [29].

**Figure 5:**
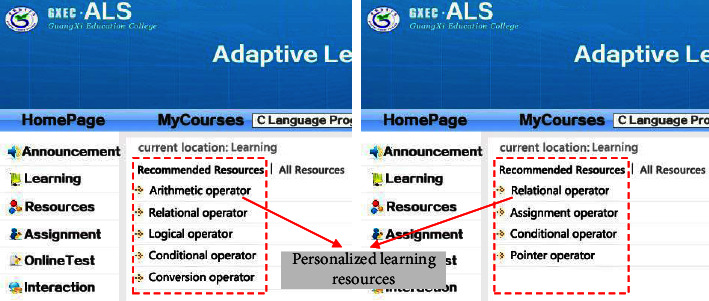
Personalized learning page of two students from group *T*1.

**Figure 6:**
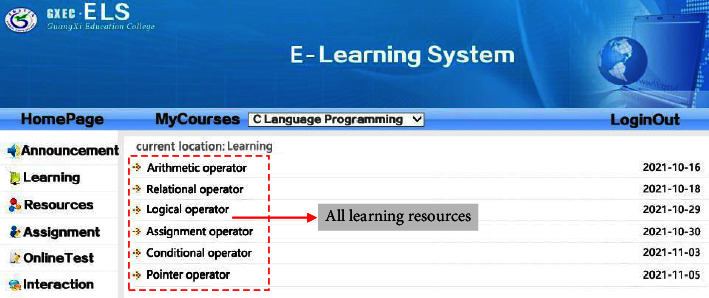
Personalized learning page of two students from group *T*2.

With the purpose of examining whether ALS can dynamically recommend personalized learning materials in accordance with the user characteristic model, an attempt is made to design and implement a series of experiments. Before the experiment, 30 students were randomly selected from two classes of educational technology major in our school as experimental samples. And then, these students are randomly divided into two groups, namely, *T*1 group (experimental group) and *T*2 group (control group). Meanwhile, account number and password are distributed to all the students in Group *T*1. During the implementation of the experiments, students in Group *T*1 and Group *T*2 are arranged in the same computer network classroom. Students in Group *T*1 are required to log in the personalized ALS that is developed in this research through the previously given account and password, and students in Group *T*2 are required to access ALS system directly through the computer browser without logging into the account. The learning contents for the two groups of students are the second chapter (operator and expression) of Programming in *C*. And, the students are required to learn all the knowledge points in the appointed chapter within 90 minutes, and take an online test afterwards. During the learning, individual students' learning pages are captured in the two groups, respectively, to examine whether the system can recommend appropriate learning resources in accordance with students' demand for personalized learning. Please see the effect from Figures 5 and 6. After the experiments, all the learning records in the system database are collected to form a sample dataset. And then, the sample data are organized and analyzed.

### 4.2. Experiment Result Analysis and Discussion

**Figure 7:**
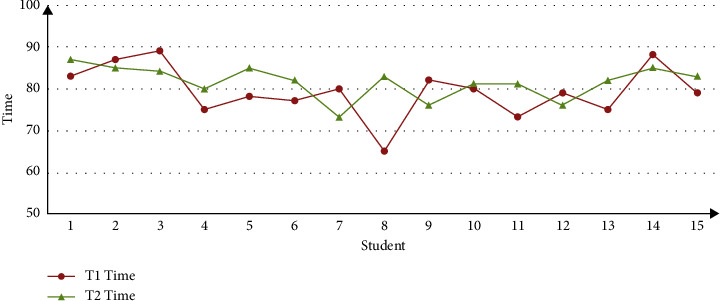
Distribution of learning length in groups *T*1 and group *T*2.

**Figure 8:**
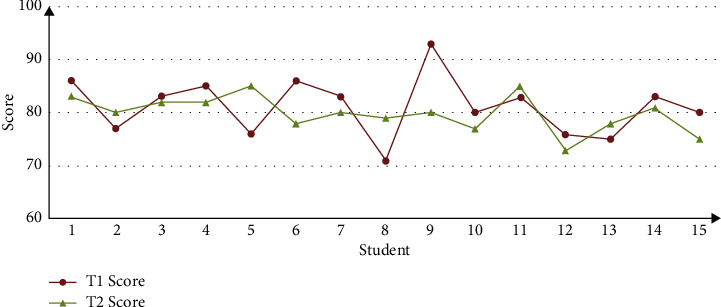
Distribution of test result in groups *T*1 and group *T*2.

With the comparison and analysis of the above experiments, it can be found that the contents of different students' learning resources in the ALS system are not the same, and all the learning resources can be browsed by clicking the button “All Resources” during the learning. After the students logged in ALS, there is no such button as “All Resources” in Figure 6, and the system presents the same learning resources to each student. This is because students in Group *T*1 are registered users, so that ALS could recommend personalized resources in accordance with the user characteristic model; on the contrary, students in Group *T*2 are not registered, and ALS cannot recommend personalized resources because it could not have access to the user characteristic model. Thus, it can be preliminarily judged that ALS developed in this research has the function of personalized recommendation to a certain extent. With the purpose of further analyzing the effect of ALS recommendation, the sample data collected from the experiments are organized and analyzed. And then, the effect of ALS recommendation is analyzed through two-dimension indexes, namely, learning length and test result. Please see the analysis result from Figures 7 and 8.

Figure 7 shows the distribution of students' online learning length in Group *T*1 and Group *T*2. This figure suggests that the time of learning is mainly between 70 and 90 mins, and the distribution track of *T*1 is generally below *T*2, which preliminarily suggests that students in *T*1 spend shorter time, while students in *T*2 spend longer time.  Figure 8 suggests the distribution of test result in Group *T*1 and Group *T*2. This figure shows that the test result of the two groups is mainly between 70 and 90, and the distribution track of *T*1 is generally above *T*2, which preliminarily suggests that students in *T*1 have better academic performance. Therefore, we believe that the reason why *T*1 group students generally get higher test scores is that they have used the ALS system, which also shows that the system has better personalized recommendation effect. With the purpose of analyzing whether there is a difference between the two groups in the distribution learning length and test result more scientifically and accurately, SPSS is conducted in *t*-test analysis. Please see the result from Table 2.

Table 2 shows that the average learning time of *T*1 and *T*2 is 78.27 and 82.87 respectively, which suggests that students in *T*1 spend shorter time than students in *T*2 on average. The standard deviation (Std. deviation) of learning time of the two groups is 6.995 and 5.012, which indicates that students in *T*2 group have relatively long concentrated performance in learning time. The significance Sig. (2-tailed) of the average time is 0.049 (less than 0.05), which suggests that there is a difference between *T*1 and *T*2 in the length of learning time. In addition, it is found through the comparison of the test result that the average score of *T*I and *T*2 is 82.67 and 79.83, respectively, which demonstrates that the average score of *T*1 is higher than the average score of *T*2. The standard deviation (Std. deviation) of test score of the two groups is 5.960 and 8.205, which indicates that students in *T*1 group have relatively high scores in the test results. The significance Sig. (2-tailed) of the average score is 0.046 (less than 0.05), which demonstrates that there is a difference between *T*1 and *T*2 in the test result. Thus, it can be inferred from Table 2 that the quality and efficiency of learning in *T*1 is superior to *T*2.

Hence, Figures 7 and 8 and Table 2 are considered comprehensively, and a conclusion is reached. The personalized ALS that is developed in this research manifests a satisfying function of recommendation, and it can recommend relatively ideal personalized learning resources in accordance with user characteristic model, which can effectively enhance learner's learning quality and efficiency to a certain extent. Based on the inference, this can be mainly attributed to the fact that ALS can analyze the current learner's demand in accordance with the user characteristic model and recommend personalized learning resources accordingly, which is conducive to learner's efficient and high-quality online learning.

## 5. Conclusions

With the purpose of implementing the effect of intelligent and personalized recommendation in accordance with user's different demands via ALS, many domestic and overseas scholars have conducted a great deal of theoretical and practical research and yielded substantial achievements. However, the current research on ALS is still in the exploratory stage, and there are a lot of fields that need to be further studied. In consideration of this, this paper intends to specifically analyze learners' traits through several dimensions, such as basic information, interest and preference, cognitive level, and learning style, and construct a user characteristic model on this basis. It is found through the research that user's demand for learning resources can be calculated through the excavation of behavior records based on the multi-dimensional modeling of learner user, through which a personalized recommendation mechanism for the learning system can be realized. In the end, an attempt is made to design and develop a prototype of the personalized ALS to test and verify the recommendation effect. And then, a series of experiments are implemented. It is suggested by the result of the experiments that ALS, which is developed, based on user characteristic model, manifests satisfying effect in recommendation, and it can dynamically recommend appropriate learning resources in accordance with learners' personalized demand, which can effectively enhance learners' learning quality and efficiency to a certain extent.

## Figures and Tables

**Table 1:** Felder-Silverman learning style model.

Dimension	Values	Description
Perception	Sensitive—intuitive	Sensitive style shows inclination towards concreteness or practice, and is good at remembering details, facts, and figures; intuitive style shows inclination towards concept or innovation, and is good at theoretical learning
Input	Visual—verbal	Visual style shows an inclination to visual expression, such as chart, picture and flow diagram, and other media information; verbal style shows an inclination towards documents and verbal expression
Processing	Active—reflective	Active style shows an inclination to practical activities, collaborative learning, activity, and exchange; reflective style shows an inclination to independent thinking and learning
Understanding	Global—sequential	Global style shows an inclination towards macroscopic blueprint and the grasp of overall concept; sequential style shows an inclination towards specific steps and gradual learning by logical sequence

**Table 2:** *T* sample test analysis result.

	Group statistics					*t*-test for equality of means	
	Group	*N*	Mean	Std. deviation	Std. error. mean	*t*	Sig. (2-tailed)
Time	*T*1	15	78.27	6.995	1.806	−2.083	0.049
	*T*2	15	82.87	5.012	1.294		

Score	*T*1	15	82.67	5.960	1.539	2.088	0.046
	*T*2	15	79.83	8.205	2.118		

## Data Availability

The data supporting the findings of this study are included within the article.
